# Novel combination of celecoxib and metformin improves the antitumor effect by inhibiting the growth of Hepatocellular Carcinoma

**DOI:** 10.7150/jca.47532

**Published:** 2020-09-14

**Authors:** Jun-Wen Hu, Bin Chen, Jie Zhang, Ya-Peng Qi, Jia-Hao Liang, Jian-Hong Zhong, Bang-De Xiang

**Affiliations:** Hepatobiliary Surgery Department, Guangxi Liver Cancer Diagnosis and Treatment Engineering and Technology Research Center, Key Laboratory for High-Incidence Tumor Prevention and Treatment, Ministry of Education, Guangxi Medical University Cancer Hospital, Nanning, China.

**Keywords:** Celecoxib, metformin, Hepatocellular carcinoma, combination, MTOR

## Abstract

**Objective:** To explore the effect of COX-2 inhibitor celecoxib in combination with metformin on the prevention of Hepatocellular carcinoma (HCC) and the mechanisms involved.

**Methods:** HCC cell lines and an HCC rat model were treated with celecoxib, metformin or a combination of both. Cell viability and tumor formation were measured.

**Results:** *In vitro* and *in vivo* studies showed that treatment with a combination of celecoxib and metformin inhibited proliferation of HCC to a greater extent than either treatment alone, by reducing the phosphorylation of MTOR.

**Conclusion:** The study suggested that celecoxib combined with metformin would be more effective for the preventing occurrence of HCC than either treatment alone and this combination of therapy is worthy of further study.

## Introduction

Hepatocellular carcinoma (HCC) is the most destructive and invasive form of liver cancers. The incidence of HCC continues to rise rapidly, ranking with the sixth most common cancer and the fourth leading cause of cancer death worldwide 1. Although the clinical diagnosis and treatment of early HCC have improved significantly, the prognosis of HCC is still very poor 2,3. In addition, highly invasive and advanced HCC responds minimally or not at all to general treatment 4-6. Therefore, there is an urgent need for new valid and well-tolerated treatment strategies. Targeted therapy has entered the field of anti-tumor therapy, bringing hope for the treatment of HCC 7-9. However, the current targeted therapy drugs generally have low tumor response rates and substantial side effects, so it is necessary to explore other types of targeted therapy against HCC.

HCC is usually the result of continuous damage and chronic inflammation. An important inflammatory mediator is the inducible gene cyclooxygenase-2 (COX-2) 10-13. COX-2 is widely expressed in various types of cancer, including liver cancer, and it promotes tumor progression and cancer cells' resistance to chemotherapy and radiation therapy 14. COX-2 is now well established as an important molecular target for anti-cancer therapy. COX-2 inhibitors have demonstrated potential therapeutic effects in HCC 15-17. Celecoxib is the COX-2 selective inhibitor which may help slow the progression of lung, breast, liver, colon and prostate tumors 18-22.

Metformin (MET) is a first-line anti-diabetic drug for the treatment of type 2 diabetes 23,24. More importantly, the anticancer effect of MET has been widely reported in recent years 25-27. In addition, MET protects the liver from chemicals or viral hepatotoxicants 28. Interestingly, it has been reported that the combined use of MET and aspirin can inhibit the growth and metastatic potential of liver cancer *in vitro* 29.

Molecular targeted therapy does bring promise for HCC, however, as in most cancers, the use of single molecular targeted drugs is unlikely to achieve long-term relief or cure in HCC, especially for advanced stages of disease 7,30. Therefore, combination therapies will be necessary, the combination of two or more anticancer drugs that target cancer in different ways has been considered a promising treatment strategy that maximizes the efficacy of the drug and reduces the side effects associated with a single component to lowest, so there seems to be reason to speculate that a combination of drugs will ultimately increase treatment benefits.

In this study, we aim to investigate the enhanced effect of Celecoxib band MET combination treatment on tumorigenesis *in vitro* and *in vivo*.

## Materials and Methods

### Reagents and drugs

The rabbit monoclonal antibody against phosphorylated mTOR (p-mTOR) (Ser2448) (49F9) was purchased from CST Corporation (USA). Celecoxib capsules and metformin hydrochloride capsules were provided by Guangxi Medical University Cancer Hospital (Nanning, China).

### Cell lines

Human HCC cell lines HepG2 and HCCLM3 were purchased from the Cell Resource Center of Shanghai Institutes for Biological Sciences (Chinese Academy of Sciences, China). The cells were cultured in a complete medium (89% DMEM + 10% fetal bovine serum + 1% penicillin and streptomycin) at 37°C in a humidified atmosphere with 5% CO_2_.

### MTT assay for cell viability

Cell viability was determined using the MTT assay (Sigma, USA) according to the manufacturer's instructions. HepG2 and HCCLM3 cells were seeded at a density of 10^4^ cells per well in 96-well plates for 24 h. Then the cells were treated for 48 h with 50 µM celecoxib, 500 µM of metformin, or the combination of 50 µM celecoxib and 500 µM metformin; control cells were treated with an equal volume of phosphate buffered saline (PBS). After treatment, 20 μL of MTT solution (5 mg/ml) was added to each well and the plates were incubated at 37 °C for 4 h. Cells were lysed by adding DMSO and the optical density of each well was measured at 450 nm. Viability was calculated as a percentage of control cells.

### Animals

A total of 98 healthy, male Sprague-Dawley (SD) rats (6 weeks old) weighing 150-200 g were provided by the Animal Experiment Center of Guangxi Medical University. Animals were maintained in a specific pathogen-free (SPF) room under laminar flow and given sterilized food and water.

### *In vivo* tumorigenesis experiment

The rats were divided into 5 groups, 10 rats were randomly selected as the normal control group, without special treatment during the experiment, all the remaining 88 rats were intraperitoneally injected with DEN dissolved with saline solution (50mg / kg, once / week for 18 weeks), then they were randomly divided into four group (n = 22 in each group) as follows: placebo group, metformin-treated (300 mg/kg) group, celecoxib-treated (100 mg/kg) group, and combination-treated (metformin + celecoxib) group. Drug treatment was administered daily for 18 weeks by oral gavage and the placebo group received an equal volume of saline solution by oral gavage. The dose of metformin and celecoxib was chosen according to previous preclinical researches and is anticipated to be innoxious. Throughout the *in vivo* experiment, normal diet was maintained for all rat groups. Body weight was measured once a week and the general behavior of the rats, such as their activity, mentality, eating, feces and hair color, were closely observed during the experiment. At the end of week 19, all the rats were sacrificed by intraperitoneal injection of 10% chloral hydrate. Liver tissues from each rat were fixed in formaldehyde for hematoxylin-eosin (HE) and immunohistochemical staining.

### Immunohistochemical staining

Paraffin sections were baked in an oven at 65 °C for 2 h. After dewaxing with xylene and dehydrating with gradient alcohol, antigen retrieval was performed by immersing sections in citric acid solution at 100 °C for 15 min. Then sections were cooled to room temperature and washed twice with PBS. Sections were treated with 3% hydrogen peroxide and incubated at 37 °C for 10 minutes, washed 3 times with PBS, and blocked with normal goat serum. The sections were then incubated overnight at 4 °C with p-mTOR antibody (working concentration 1: 100). Tissue slices were washed with PBS three times, incubated with biotinylated secondary antibody (Zhongshan Golden Bridge Biotechnology Company, Beijing China) for 30 min at room temperature. After another PBS wash, sections were incubated for 30 min with streptavidin-peroxidase complex (Zhongshan Golden Bridge Biotechnology Company, Beijing China). The color reaction was developed with diaminobenzidine, finally, sections were counterstained with hematoxylin. Tumors with > 25% p-mTOR positive cells were deemed positive for antigen expression. Pathological diagnoses of tissue sections were determined by two experienced pathologists blinded to treatment.

### Statistical analyses

All data during the experiment were analyzed by SPSS22.0 software. Quantitative data are described by means ± standard deviation. The comparison of the means between groups was performed by one-way analysis of variance, followed by the least significant difference (LSD) method; the statistics of the composition ratio or categorical variable rate were tested using the chi-squared test or Fisher's exact probability method. Differences associated with *P* <0.05 were considered statistically significant.

## Results

### Celecoxib and metformin combination treatment significantly reduces HCC cell survival *in vitro*

**Figure 1:**
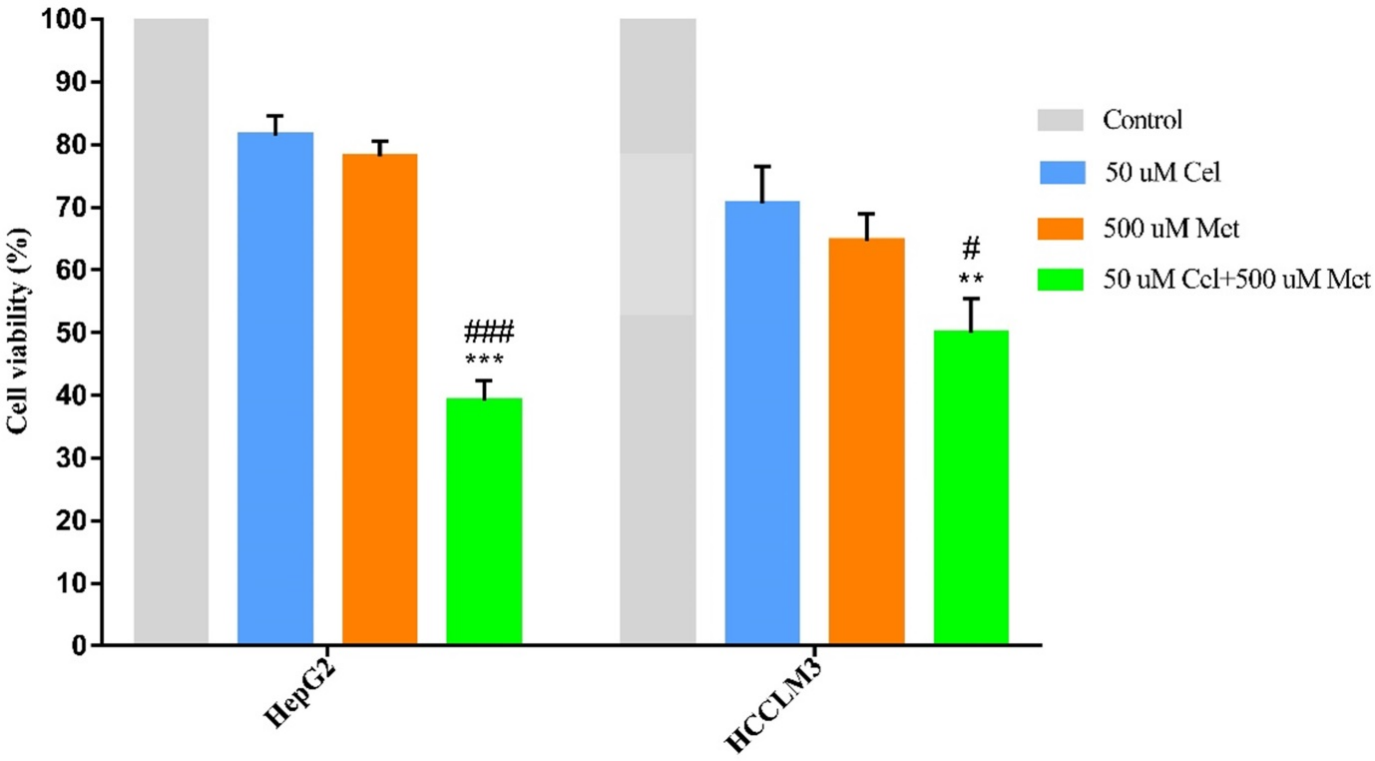
Effect of celecoxib, metformin and their combination on cell survival in HCC cell lines. HepG2 and HCCLM3 cells were treated with the specified dosage of celecoxib and metformin either alone or in combination for 48h. Then MTT assay was used to measure cell viability. Data are represented as a percent of control cells and are the mean ± SD of three independent experiments. **P < 0.01, ***P <0.001 compared with celecoxib alone; ^#^*P* < 0.05, ^###^*P* <0.001 compared with metformin alone.

The combination treatment of celecoxib and metformin displayed significantly higher cytotoxicity than either drug alone (**Figure 1, Table 1**). Cell survival was similar after celecoxib or metformin treatment alone.

### Combination treatment significantly reduces HCC tumor formation in rats

**Figure 2:**
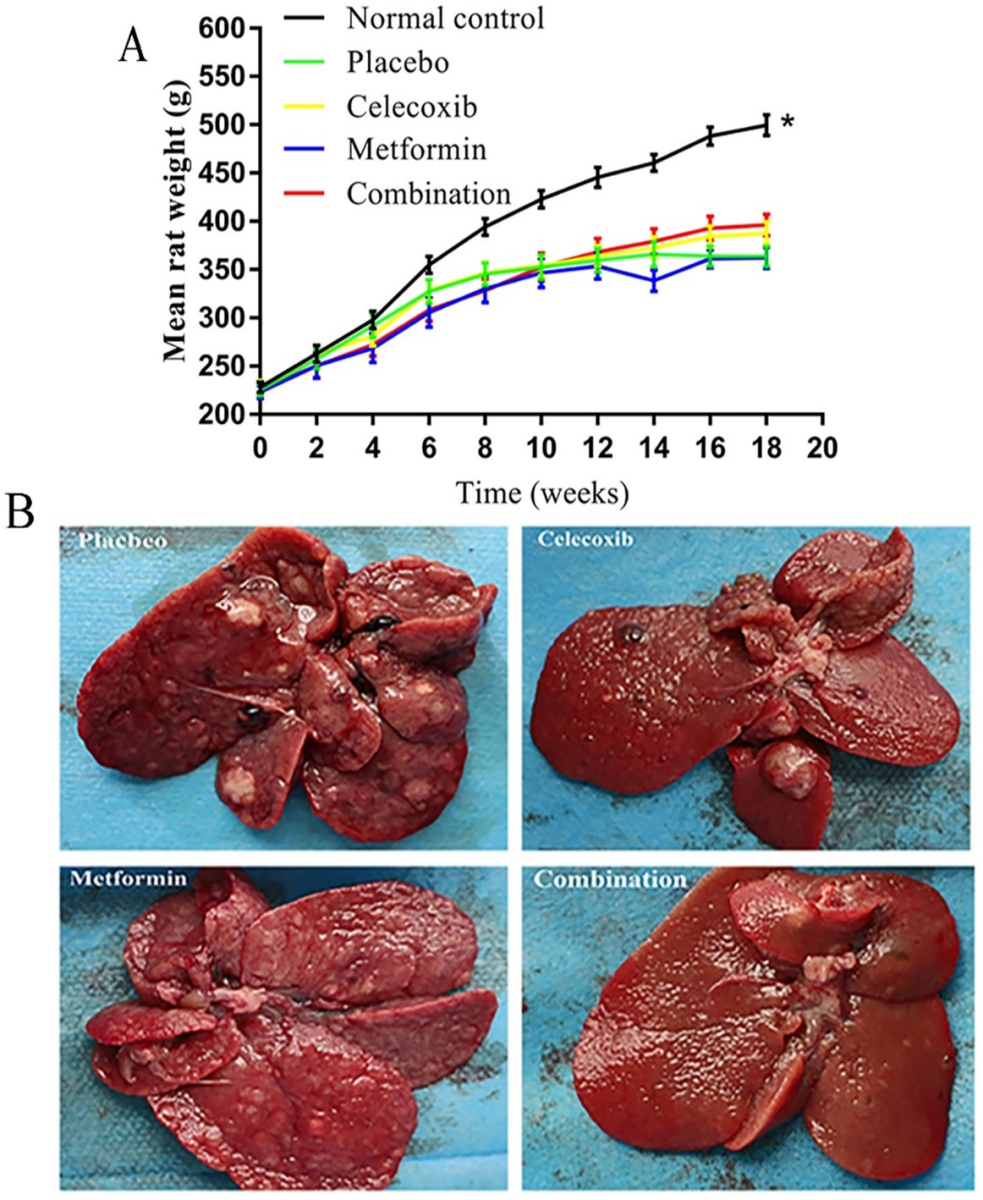
Effects of celecoxib and metformin alone and in combination on the growth of hepatocellular carcinoma in rats. (**A**) Quantification of rat weights in each group measured over time. *P<0.05 versus control. (**B**) Photographs of tumors in representative livers isolated from rats in each treatment group at the end of the 18 weeks of treatment.

The rats in the normal control group gained significantly more weight over time than the treatment groups (**Figure 2A**). Notably, the rats treated with a combination of drugs gained more weight than other experimental rats beginning at week 12; however, the differences failed to reach statistical significance. Before rats were sacrificed at week 19, a small number of rats in each experimental group died prematurely due to intestinal obstruction, gastrointestinal perforation, and intestinal necrosis caused by side effects.

Based on gross anatomy and HE staining, the final tumor formation rate in each group was as follows: placebo group, 65.0%; celecoxib group, 36.8%; metformin group, 42.9%; and combination drug group, 17.6% (**Figure 2B, Table 2**). Combination treatment led to significantly lower tumor formation rate than placebo.

### Combination treatment reduces p-mTOR in cancerous liver tissue

**Figure 3:**
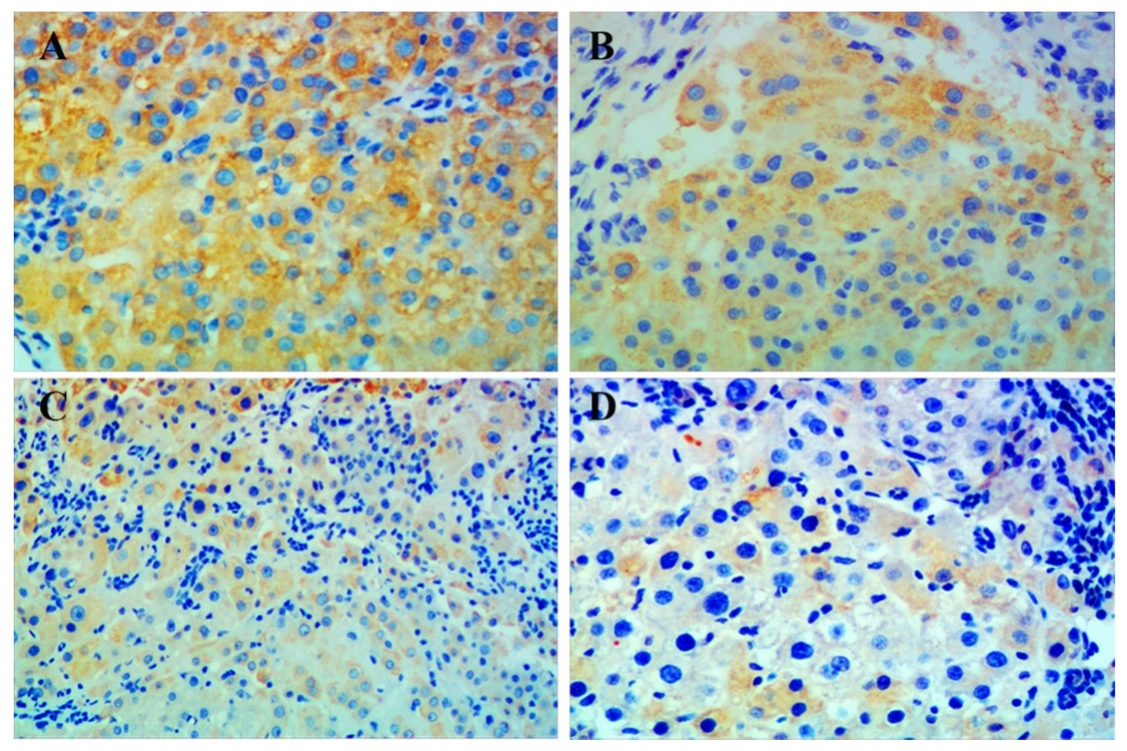
Immunohistochemical staining for p-mTOR in liver tissue. Representative images of liver tissue isolated from rats with hepatocellular carcinoma and treated with (**A**) placebo, (**B**) celecoxib, (**C**) metformin, or (**D**) combined metformin and celecoxib. Magnification × 200.

Immunohistochemistry showed that p-mTOR was highly increased in liver tissue from all rats with HCC. The group given combination treatment showed the lowest positivity of p-mTOR among all groups. However, positive rates between the groups were not statistically significant, which may be due to the small number of samples (**Figure 3, Table 3**).

## Discussion

HCC is an extremely complex tumor that requires effective treatment through multi-pronged approach. Finding more effective drugs to treat HCC is of great significance. Monotherapy for cancer may produce chemotherapy resistance, whereas a great number of researches have revealed that combined treatment can enhance antitumor effects 31-34. The multi-target-based approach appears more appropriate for treating HCC, implying that combination therapy should be more effective.

Celecoxib is a first-line non-steroidal anti-inflammatory drug and a potent inhibitor of COX-2. It has shown anti-cancer activity in many different types of cancer cells and animal models including liver cancer 35-40. Many previous studies have found that celecoxib can not only inhibit the proliferation of a variety of tumors, but also induce apoptosis of tumor cells. Moreover its inhibition of cancer cells appears to be time and concentration dependent 41-43. Celecoxib is effective against human epithelial cell type tumors, the US Food and Drug Administration (FDA) has approved oral celecoxib for patients with familial colon adenoma polyps to prevent colon cancer 44.

Metformin is a widely used in the clinic as an oral hypoglycemic drug. Recently, the therapeutic effect of metformin on tumors has attracted attention. Many studies have shown that metformin can also treat tumors, such as those found in lung, breast, colon and prostate 45-47. Metformin may reduce the risk of HCC in patients with diabetes, implying that metformin may also have a therapeutic effect against HCC 48. The proposed mechanism underlying this therapeutic effect is that metformin inhibits cell growth and promotes apoptosis in a dose-dependent manner 49.

Previous studies have determined the effects of either metformin or celecoxib together with standard chemotherapeutics. When metformin is combined with gefitinib, it can produce stronger cytotoxicity in lung squamous cancer cells, and enhance the growth inhibition of lung cancer cells 50. Metformin can enhance the sensitivity of ovarian cancer cell lines to carboplatin when given in combination 51. Together with ionizing radiation, metformin enhances cytotoxicity and inhibits DNA repair in liver cancer cells 52. Similarly, celecoxib in combination with Bacillus Calmette-Guerin immunotherapy is more effective against urothelial cell carcinoma than intravesical therapy alone 53. Celecoxib combined with fluvastatin is more effective against HCC than either treatment alone 54. The combination of celecoxib with the multikinase inhibitor sorafenib produces a synergistic apoptotic effect against liver cancer cells 55. These studies have shown that combination medication is better than single medication in treating many types of cancer cells.

A previous study investigating the combined application of metformin and celecoxib found that together they synergistically inhibited cell proliferation in a concentration-dependent manner. Since this increase in cytotoxicity does not increase DNA damage, this combination can be used to inhibit the growth of malignant cells without any genotoxic or mutational effects at the cellular level 56. Therefore, combined molecular targeted therapy can be used for certain types of advanced malignancies. Metformin combined with celecoxib may be a viable option to treat or prevent tumors, especially in obese and diabetic patients.

In order to verify whether the combined use of metformin and celecoxib can effectively prevent the occurrence of HCC, we first tested the combined effects *in vitro* by MTT assay. Then we established an HCC rat model to explore the antitumor effects. The results showed that treatment with celecoxib or metformin alone only produced negligible decreases in tumor formation rate. In contrast, combined medication significantly reduce tumor formation rate in rats. Our research shows that the combination of drugs is more effective than either drug alone in inhibiting the occurrence of liver cancer.

mTOR is a key downstream gene in many signaling pathways, and its increased phosphorylation level can promote tumor cell growth and development, conversely reducing the phosphorylation level of mTOR can inhibit these pathways and then restrain the tumorigenesis and development of tumors 57,58. Celecoxib or metformin in combination with other drugs reduces the phosphorylation level of mTOR by inactivating the PI3K/Akt/mTOR or AMPK/mTOR signaling pathways, resulting in tumor inhibition 59-61.

In order to further study the anti-hepatic mechanism of celecoxib combined with metformin against HCC, we used immunohistochemical staining to detect p-mTOR in liver tissue of each group of rats, and found that the positive expression rate of p-mTOR in the combination group was lowest. Our results suggested that celecoxib combined with metformin may synergistically suppress mTOR-related signaling pathways by reducing mTOR phosphorylation, thereby inhibiting tumorigenesis.

We have confirmed for the first time *in vitro* and vivo experiments that celecoxib combined with metformin can synergistically inhibit the occurrence of liver cancer, and we have initially revealed the mechanism. However, our findings have some limitations. First, the number of our experimental samples was relatively small. A small proportion of rats in the experimental groups died from intestinal obstruction, gastrointestinal perforation, and intestinal necrosis due to drug side effects. Second, we studied only one mechanism that may underlie the therapeutic efficacy of the combined therapy. Given the complexity of molecular pathways in cancer development and pharmaceutical regulation, further studies should explore other potential mechanisms.

In summary, our results suggest that celecoxib combined with metformin may inhibit the occurrence and development of liver cancer, as demonstrated in rat models of HCC and appears to involve synergistic inhibition of mTOR-related signaling pathways. Larger studies are required to assess and maximize the safety and efficacy of celecoxib and metformin in the treatment of HCC, and to explore the molecular mechanism of synergistic inhibition of tumors before clinical application.

## Figures and Tables

**Table 1:** Cell survival of drug-treated hepatocellular carcinoma cell lines, as a percentage of survival in control cells

Cell line	50 µM Cel	500 µM Met	50 µM Cel + 500 µM Met
HepG2	81.43±3.12	78.07±2.54	39.12±3.16^a^
HCCLM3	70.59±5.95	64.65±4.21	49.97±5.43^b,c^

Values are presented as mean ± SD; ^a^ *P* <0.001 compared to celecoxib or metformin treatment alone; ^b^ *P* < 0.01 compared to celecoxib treatment alone; ^c^ *P* < 0.05 compared to metformin treatment alone; Cel, celecoxib; Met, metformin.

**Table 2:** Comparison of hepatocellular carcinoma tumor formation in rats after drug treatment

Oncogenesis	placebo (n=20)	celecoxib (n=19)	metformin (n=14)	combination (n=17)
No	7 (35)	12 (63.2)	8 (57.1)	14 (82.4)^a^
Yes	13 (65)	7 (36.8)	6 (42.9)	3 (17.6)

Values are shown as n (%); ^a^ *P*<0.05 compared to the placebo group.

**Table 3:** Comparison of p-mTOR positivity in liver sections from rats with induced hepatocellular carcinoma after drug treatment

Positivity	placebo (n=18)	celecoxib (n=15)	metformin (n=12)	combination (n=14)
No	7 (38.9%)	7 (46.7%)	7 (58.3%)	9 (64.3%)
Yes	11 (61.1%)	8 (53.3%)	5 (41.7%)	5 (35.7%)

Values are expressed as n (%).

## Abbreviations

HCC: Hepatocellular carcinoma
COX-2: cyclooxygenase-2
MET: Metformin
DEN: diethylnitrosamine
SPF: specific pathogen free
HE: hematoxylin-eosin
PBS: phosphate buffered saline
OD: Optical density
BCG: Bacillus Calmette-Guerin
